# Tumor Multifocality is a Significant Risk Factor of Urinary Bladder Recurrence after Nephroureterectomy in Patients with Upper Tract Urothelial Carcinoma: A Single-Institutional Study

**DOI:** 10.3390/diagnostics10040201

**Published:** 2020-04-03

**Authors:** Chuan-Shu Chen, Jian-Ri Li, Shian-Shiang Wang, Cheng-Kuang Yang, Chen-Li Cheng, Chi-Rei Yang, Yen-Chuan Ou, Hao-Chung Ho, Chia-Yen Lin, Sheng-Chun Hung, Cheng-Che Chen, Shu-Chi Wang, Kun-Yuan Chiu, Shun-Fa Yang

**Affiliations:** 1 Institute of Medicine, Chung Shan Medical University, Taichung 402, Taiwan; r2060d@gmail.com (C.-S.C.); fisherfishli@yahoo.com.tw (J.-R.L.); sswdoc@yahoo.com.tw (S.-S.W.); cheng20011@gmail.com (C.-L.C.); lcyhank.tw@gmail.com (C.-Y.L.); weshong1118@gmail.com (S.-C.H.); 2 Division of Urology, Department of Surgery, Taichung Veterans General Hospital, Taichung 407, Taiwan; yangck@vghtc.gov.tw (C.-K.Y.); brainhcho@gmail.com (H.-C.H.); bigkuky2001@yahoo.com.tw (C.-C.C.); farmgy1980@yahoo.com.tw (S.-C.W.); 3 Department of Medicine and Nursing, Hungkuang University, Taichung 433, Taiwan; 4 Department of Applied Chemistry, National Chi Nan University, Nantou 545, Taiwan; 5 Department of Urology, China Medical University Hospital, Taichung 404, Taiwan; cryang@mail.cmuh.org.tw; 6 Department of Urology, Tung’s Taichung MetroHarbor Hospital, Taichung 433, Taiwan; ycou228@gmail.com; 7 Department of Urology, Chan Bin Show-Chwan Memorial Hospital, Changhua 505, Taiwan; 8 Department of Medical Research, Chung Shan Medical University Hospital, Taichung 402, Taiwan

**Keywords:** urinary bladder, recurrence, nephroureterectomy, upper tract urothelial carcinoma, tumor multifocality

## Abstract

The purpose of this study was to identify the significant risk factors of urinary bladder recurrence (UBR) after nephroureterectomy (NUx) in patients with upper tract urothelial carcinoma (UTUC). A total of 550 patients diagnosed with UTUC between January 2001 and December 2015 were included in this retrospective study. The median age of our patients was 68 (range 24–93) and the median follow-up time after NUx was 40.3 months (range 8–191). The most important censored point of this study was the first episode of UBR. Of the 550 patients, UBR occurred in 164 patients (29.8%). One hundred and forty-two (86.6%) patients with UBR were identified within two years after NUx for UTUC, with the median time interval between NUx and UBR being 8.4 months (range 3–59.8). Through univariate analysis, the positive surgical margin (*p* = 0.049) and tumor multifocality (*p* = 0.024) were both significant prognostic factors for UBR-free survival after NUx in patients with UTUC. However, only tumor multifocality (*p* = 0.037) remained a significant prognostic factor by multivariate analysis. In conclusion, tumor multifocality is a significant risk factor of UBR after nephroureterectomy in patients with upper tract urothelial carcinoma.

## 1. Introduction

According to published reports, upper tract urothelial carcinoma (UTUC), involving the renal pelvis, renal calyces or ureter was found in less than 5% of all urothelial carcinoma cases [1]. However, in Taiwan the proportion of UTUC in all urothelial carcinoma cases was 40.2%, with the incidence of UTUC being obviously higher than that in Western countries [2,3].

Radical nephroureterectomy (NUx) with excision of the urinary bladder cuff is the standard treatment for a patient with localized UTUC. However, postoperative tumor recurrences, including urinary bladder recurrence (UBR), local retroperitoneal recurrence and contralateral UTUC, were relatively high [4]. Among these types of recurrences, UBR was the most common, with an incidence rate of approximately 30%–40% [5,6,7,8]. The main hypotheses in explaining the mechanisms of UBRs were intraluminal seeding and field change with carcinogenic events and early genetic alternations [9]. Many previous studies have concentrated on the risk factors of UBR in patients with UTUC after NUx but their results were diverse [5,6,8,10]. UTUC is an endemic cancer in Taiwan [2]. Therefore, following strict patient-enrollment criteria, we attempted to retrospectively select our UTUC Taiwanese patients, who had undergone NUx in a single institute to pool their clinicopathological information to form a data bank. Through analysis of this data bank, we then determined the possible significant and predictive factors of postoperative UBR in patients with UTUC.

## 2. Materials and Methods

### 2.1. Description of Enrolled Subjects

This retrospective study was approved by our institutional review board (No. CE13240; approved on 3 September 2013) prior to the start of the study. Between January 2001 and December 2015, 668 patients with UTUC underwent NUx in our hospital. One hundred and eighteen patients were excluded, including 40 patients with bilateral UTUC who had undergone bilateral NUx, eight patients with clinically metastatic UTUC, 36 patients with a previous history of urothelial carcinoma of the urinary bladder, 15 patients without any postoperative cystoscopic follow-up and 19 patients who had undergone a concomitant transurethral resection of the bladder tumor or a cystectomy. In the end, this study enrolled a total of 550 patients. All enrolled patients received adequate preoperative clinical staging, including a ureteroscopic biopsy, abdominal and pelvic computed tomography (CT)/ magnetic resonance imaging (MRI) and a chest X-ray. Although nephroureterectomy with excision of the urinary bladder cuff for all enrolled patients was performed by different urosurgeons, all procedures for this operation were standardized in our institute, whether it was the open or laparoscopic/retroperitoneoscopic method that was implemented. However, the extent of lymph node dissection was not standardized and was decided by each urosurgeon, as it was based upon preoperative images and intraoperative findings. The pathological evaluation of all postoperative specimens was also standardized. Basic patient demographics, including gender, age, body mass index (BMI), preoperative renal function, performance status and smoking status were recorded retrospectively. Postoperative tumor characteristics and pathological stages were diagnosed by two genitourinary pathologists according to the 2016 World Health Organization classification. The 2002 TNM classification was only adopted for tumor grading. After NUx, the patients who had reached a stage higher than pT2N0M0 were advised to receive systemic chemotherapy, including methotrexate, an epirubicin and cisplatin/carboplatin (MEC) regimen or a gemcitabine and cisplatin/carboplatin (GC) regimen. Postoperative follow-up for those patients with UTUC included a periodic history and physical examinations, urine cytology, cystoscopy, pyelography, CT/MRI and a chest X-ray. The cystoscopic follow-up protocol included performing cystoscopy every three months for two years, every six months for the following three years, and then once annually thereafter.

### 2.2. Statistical Analysis

The most important censored point of this study was the first episode of UBR. UBR-free survival was defined as the interval between the date of NUx and the date of the first episode of UBR. Survival data was analyzed using both the Kaplan–Meier method and the logrank test. Univariate and multivariate analyses by Cox’s proportional hazards model were used to determine the relevance between each of the clinicopathological factors and UBR. P-values less than 0.05 were defined as statistically significant. All statistical analyses were performed with SPSS (Statistical Package for the Social Sciences, version 22.0, IBM, NY, USA).

## 3. Results

**Figure 1:**
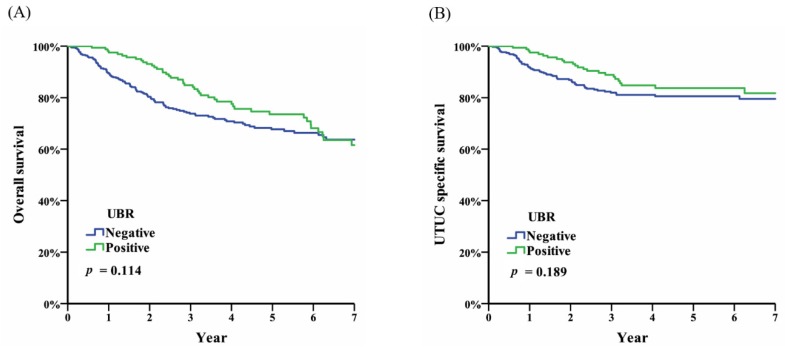
(**A**) Overall survival of upper tract urothelial carcinoma (UTUC); and (**B**) UTUC-specific survival stratified by urinary bladder recurrence (UBR).

Of the 550 patients with UTUC after NUx that we had enrolled, 237 (43.1%) were male and 313 (56.9%) were female. One hundred and sixty-four patients (29.8%) had UBR and 142 (86.6%) of the 164 patients were identified within two years, with the median time interval between NUx and UBR being 8.4 months (range 3–59.8). The median age was 68 years (interquartile range 59–75.3) and the median follow-up time was 40.3 months (interquartile range 23.6–71.4). Two hundred sixty (47.3%) patients had a normal BMI (18.5–24 kg/m^2^), while 240 (43.6%) patients had abnormal renal function (serum creatinine > 1.4 mg/dL). Seventy-two (13.1%) patients had a history of uremia. One hundred and sixteen (21.1%) patients received conventional open-method NUx. A transurethral resection of the bladder tumor was performed for all patients with UBR. All pathological reports showed superficial urothelial carcinoma of the urinary bladder (T1) without any muscle-invasive bladder cancer. Other demographic information, tumor characteristics and pathological results are summarized in Table 1. Whether UBR was diagnosed or not, the overall survival and UTUC-specific survival was not impacted (Figure 1).

**Figure 2:**
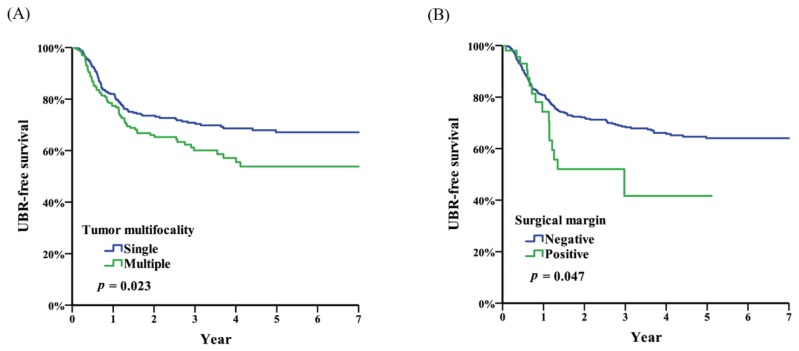
Urinary bladder recurrence (UBR)-free survival stratified by (**A**) surgical margin; and (**B**) tumor multifocality.

We used 15 clinicopathological parameters to analyze which parameters could be statistically significant to UBR. Through the use of univariate analysis, surgical margin (*p* = 0.049, HR = 1.68) and tumor multifocality (*p* = 0.024, HR = 1.43) were significant predictors for UBR. However, multivariate analysis using the Cox regression model disclosed that the only predictive parameter is tumor multifocality (*p* = 0.037, HR = 1.40) (Table 2). Figure 2 demonstrates UBR-free survival, according to the surgical margin status, and tumor multifocality for all patients.

## 4. Discussion

UBR is the most common type of recurrence in patients with UTUC after NUx [5,6,7,8]. In our study, we have summarized our 15-year experience in Taiwan in dealing with this affliction. Of the 550 enrolled patients with UTUC after NUx, 164 (29.8%) had UBR. The pathophysiology of UBR was not clearly elucidated. There were two hypotheses to explain the possible mechanism of UBR in patients with UTUC after NUx. The first one, the “field change” hypothesis, implies that carcinogenic events and early genetic alternations occurred in the microenvironment of the entire urothelium. The second hypothesis, known as “intraluminal seeding”, stated that active cancer cells traveled and implanted themselves into the benign urothelium [9]. These two mechanisms can co-exist, however, we wanted to know which one played the major role. We attempted to apply these two mechanisms in order to explain the contralateral recurrence. Contralateral recurrence was found in 30 (5.5%) of the patients in our study, and was mainly caused by the mechanism of field change. The mechanism of intraluminal seeding may play a minimal role in contralateral recurrence, because active cancer cells easily travel downstream, while movement upstream is difficult. We assumed the effect of intraluminal seeding would be obviously stronger than field change on the recurrences, because the incidence of UBR (29.8%) was much higher than that of contralateral recurrence (5.5%).

Seisen et al. [11] reported on a systemic review and meta-analysis concerning the clinicopathological factors of UBR in patients with UTUC after NUx. This review article involved 18 studies and more than 8000 patients. It concluded that diverse predictors, including three patient-specific predictors, five tumor-specific predictors and three treatment-specific predictors were statistically significant to UBR. The positive finding in our study, tumor multifocality (*p* = 0.037, HR = 1.40), was also included in their tumor-specific predictors. When we looked into the forest plot of meta-analyses of tumor multifocality in their article, we found six positive studies [6,8,12,13,14,15] and tumor multifocality (HR = 1.61, 95% CI 1.27–2.03; *p* < 0.001) was a significant predictor of UBR. When compared with other significant predictors from their article, tumor multifocality was found to be approved by the most studies, so this factor could be one of more importance. With the same patient ethnicity and similar inclusion and exclusion criteria, Liu et al. [14] revealed that their incidence of UBR (30.2%) was close to our data findings. In their study, tumor multifocality was also an unignorable parameter of UBR.

The approach methods of NUx are an important issue for UBR. The approach methods could be divided into transperitoneal open, retroperitoneal open, laparoscopic and retroperitoneoscopic w/o robotic-assisted methods. Based upon published data [6,16,17], NUx involving the laparoscopic method showed a higher incidence of UBR. However, our results do not show a significantly different incidence between the open and laparoscopic methods. A possible explanation for this may be the early clipping of the distal ureter. This procedure was standardized and performed during the initial part of NUx in our hospital.

The status of surgical margins was another significant predictor of UBR, as concluded by Seisen et al. [11]. However, their review article included only two positive studies. Our results show that a positive surgical margin (*p* = 0.101, by multivariate analysis) was not a predictive parameter of UBR. Similar results were also found in the study from Zou et al. [16]. Therefore, we required more data in our study in order to make a final conclusion.

In our clinical practice, a high recurrence rate was discovered when we did transurethral resections of urinary bladder urothelial carcinoma with the existence of carcinoma in situ (CIS). The incidence of CIS in our cohort was 14.7% and this parameter was not found to be a significant predictor (*p* = 0.169 by univariate analysis) in our study. Although some large studies have supported the existence of CIS [5,8,18] as a significant predictor of UBR, Seisen et al. [11] concluded that the existence of CIS was not a significant parameter of UBR, due to the heterogeneity of their included data. According to the mechanism of intraluminal seeding and implantation, we inferred that the heterogeneity may be caused by the site of CIS. We have hypothesized that the site of CIS was closer to the urinary bladder, and therefore more patients with UBR would be detected. However, none of these studies mentioned the site of CIS as being a cause.

Prophylactic intravesical chemotherapy to prevent UBR in patients with UTUC after NUx has been recommended throughout many studies [19,20]. In our cohort, nearly all the enrolled patients never received any prophylactic intravesical chemotherapy. In the past two years, more of the urosurgeons in our hospital have now followed this recommendation.

Close postoperative follow-up remains the best policy for UBR. According to our results, the incidence of UBR (29.8%) was high, with 86.6% of UBR occurring within two years. The median time interval between NUx and UBR was 8.4 months. Therefore, our follow-up protocol was to perform cystoscopy every three months for the first two years, every six months for the following three years and then once annually thereafter. This protocol was similar to most studies [7,8,13,14]. We must emphasize that if cystoscopy is performed less frequently in the first two years after NUx, there may be a delayed diagnosis of UBR, and furthermore an occurrence of muscle-invasive bladder cancer. Fortunately, all patients with UBR had only a superficial form of the disease (T1). However, Kim et al. [7] demonstrated that their incidence of UBR was 40.9% and 7.5% of first UBR patients were diagnosed with muscle-invasive bladder cancer. Their cystoscopic follow-up protocol was the same as ours. The authors concluded that the only risk factor regarding these muscle-invasive UBRs was a previously diagnosed or concomitant bladder cancer. Such patients were excluded from our study, and therefore muscle-invasive bladder cancer was not found in our patients with UBR.

Our study involved a larger sample size and strict inclusion/exclusion criteria, and was completed in a single institute. Although NUx was performed by several urosurgeons, all of them followed the same standards of procedure for NUx. Additionally, they followed the same protocols for postoperative follow-up. However, limitations within our study still existed. Because this was a retrospective study, selection bias could not be avoided, in spite of strict inclusion/exclusion criteria. When we asked our patients for personal information such as their smoking status, recall bias appeared. Therefore, some studies stated that this factor was a possible confounder [7,14]. There still remain some new ideas regarding further study that should be addressed. In the future, we would record the site of CIS in order to have that analyzed and we would also enroll more patients who had undergone intravesical chemotherapy to compare their data with the current results.

## 5. Conclusions

In conclusion, UBR is the most common type of recurrence in patients with UTUC after NUx. In our study, we included 550 patients over a 15-year period and, by analyzing their clinicopathological data, we concluded that tumor multifocality was the only significant parameter for predicting UBR. According to our data, whether UBR existed or not did not impact overall survival or cancer-specific survival of UTUC. Additionally, the late detection of UBR or its recurrence, resulting in muscle-invasive bladder cancer, would negatively impact a patient’s survival. Therefore, a postoperative cystoscopic follow-up protocol (every three months for the first two years, every six months for the following three years, and then once annually thereafter) is critical for avoiding a delayed diagnosis or muscle-invasive bladder cancer due to UBR.

## Figures and Tables

**Table 1:** Clinicopathological characteristics of 550 patients with UTUC after NUx.

Variables	*N*	%	Variables	*N*	%
**Gender**			**Tumor location**		
Male	237	(43.1%)	calyx	129	(23.5%)
Female	313	(56.9%)	renal pelvis	338	(61.5%)
**Age**			proximal ureter	167	(30.4%)
≤65	220	(40.0%)	middle ureter	109	(19.8%)
>65	330	(60.0%)	distal ureter	120	(21.8%)
**Performance status (ECOG)**			**Tumor multifocality**		
0	57	(10.4%)	Single	338	(61.5%)
1	384	(69.8%)	Multiple	212	(38.5%)
2	101	(18.4%)	**Surgical margin**		
3	6	(1.1%)	Negative	497	(90.4%)
4	2	(0.4%)	Positive	53	(9.6%)
**Body mass index (BMI)**			**Pathological TNM stage**		
<18.5	25	(4.5%)	T1 N0 M0	259	(47.1%)
18.5–24	260	(47.3%)	T2 N0 M0	78	(14.2%)
24–27	170	(30.9%)	T3 N0 M0	145	(26.4%)
≥27	95	(17.3%)	T4 N0 M0	12	(2.2%)
**Smoking status**			Tany N1 M0	18	(3.3%)
Never	391	(71.1%)	Tany N2/3 M0	16	(2.9%)
Current	76	(13.8%)	Tany Nany M1	22	(4.0%)
Former	83	(15.1%)	**Tumor grade**		
**Preoperative renal function (mg/dL)**			G1	16	(2.9%)
≤1.4	310	(56.4%)	G2	182	(33.1%)
>1.4	240	(43.6%)	G3	352	(64.0%)
**History of uremia**			**Concomitant CIS**		
Negative	478	(86.9%)	Negative	469	(85.3%)
Positive	72	(13.1%)	Positive	81	(14.7%)
Renal transplantation (−)	65	(90.3%)	**Lymphovascular invasion**		
Renal transplantation (+)	7	(9.7%)	Negative	430	(78.2%)
**Side**			Positive	120	(21.8%)
Right	244	(44.4%)	**Recurrence**		
Left	306	(55.6%)	Local		
**Surgical modality**			Negative	506	(92.0%)
Open	116	(21.1%)	Positive	44	(8.0%)
Laparoscopy	417	(75.8%)	UB		
Retroperitoneoscopy	17	(3.1%)	Negative	386	(70.2%)
			Positive	164	(29.8%)
			Contralateral		
			Negative	520	(94.5%)
			Positive	30	(5.5%)

**Table 2:** Univariate and multivariate analyses of clinicopathological parameters predicting UBR.

	**Univariate**			**Multivariate**		
	**Hazard Ratio**	**(95%CI)**	***p***	**Hazard Ratio**	**(95%CI)**	***p***
**Gender**						
Female	1.00	(Reference)				
Male	1.22	(0.90–1.66)1.66)	**0.203**			
**Age**						
≤65	1.00	(Reference)				
>65	0.99	(0.72–1.35)1.35)	**0.941**			
**Performance status (ECOG)**						
0	1.00	(Reference)				
1	1.53	(0.88–2.65)2.65)	**0.134**			
2	1.17	(0.61–2.26)2.26)	**0.639**			
3	-					
4	-					
**BMI**						
<18.5	1.00	(Reference)				
18.5–24	1.25	(0.54–2.88)2.88)	**0.595**			
24–27	1.60	(0.69–3.69)3.69)	**0.272**			
≥27	0.86	(0.35–2.12)2.12)	**0.737**			
**Smoking status**						
Never	1.00	(Reference)				
Current	1.20	(0.78–1.83)1.83)	**0.408**			
Former	1.10	(0.66–1.83)1.83)	**0.708**			
**Preoperative renal function (mg/dl)**						
≤1.4	1.00	(Reference)				
>1.4	1.25	(0.92–1.70)1.70)	**0.157**			
**History of uremia**						
Negative	1.00	(Reference)				
Positive	1.46	(0.97–2.20)2.20)	**0.073**			
**Surgical modality**						
Open	1.00	(Reference)				
Laparoscopy	1.00	(0.68–1.46)1.46)	**0.987**			
Retroperitoneoscopy	1.17	(0.49–2.80)2.80)	**0.722**			
**Tumor location**						
Without distal ureter tumor	1.00	(Reference)				
With distal ureter tumor	1.37	(0.97–1.95)1.95)	**0.078**			
**Tumor multifocality**						
Single	1.00	(Reference)		1.00	(Reference)	
Multiple	1.43	(1.05–1.95)	0.024 *	1.40	(1.02–1.91)	0.037 *
**Surgical margin**						
Negative	1.00	(Reference)		1.00	(Reference)	
Positive	1.68	(1.00–2.83)	0.049 *	1.55	(0.92–2.62)	**0.101**
**Pathological TNM stage**						
T1 N0 M0	1.00	(Reference)				
T2 N0 M0	0.98	(0.63–1.52)1.52)	**0.938**			
T3 N0 M0	0.88	(0.61–1.27)1.27)	**0.498**			
T4 N0 M0	0.78	(0.11–5.60)5.60)	**0.803**			
Tany N1 M0	0.76	(0.28–2.06)2.06)	**0.586**			
Tany N2/3 M0	-					
Tany Nany M1	0.22	(0.03–1.57)	**0.131**			
**Tumor grade**						
G1	1.00	(Reference)				
G2	0.80	(0.37–1.75)1.75)	**0.585**			
G3	0.68	(0.31–1.46)1.46)	**0.317**			
**Concomitant CIS**						
Negative	1.00	(Reference)				
Positive	1.33	(0.89–1.98)1.98)	**0.169**			
**Lymphovascular invasion**						
Negative	1.00	(Reference)				
Positive	1.12	(0.76–1.65)1.65)	**0.566**			

Cox proportional hazard regression. * *p* < 0.05,
